# Heterologous Expression of Toxins from Bacterial Toxin-Antitoxin Systems in Eukaryotic Cells: Strategies and Applications

**DOI:** 10.3390/toxins8020049

**Published:** 2016-02-19

**Authors:** Chew Chieng Yeo, Fauziah Abu Bakar, Wai Ting Chan, Manuel Espinosa, Jennifer Ann Harikrishna

**Affiliations:** 1 Biomedical Research Centre, Faculty of Medicine, Universiti Sultan Zainal Abidin, Medical Campus, Jalan Sultan Mahmud, 20400 Kuala Terengganu, Malaysia; 2 Centre for Research in Biotechnology for Agriculture (CEBAR) and Institute for Biological Sciences, Faculty of Science, University of Malaya, 50603 Kuala Lumpur, Malaysia; afung87@gmail.com; 3 Molecular Microbiology and Infection Biology, Centro de Investigaciones Biológicas, Consejo Superior de Investigaciones Cientificas, Ramiro de Maeztu 9, 28040 Madrid, Spain; chanyting@hotmail.com (W.T.C.); mespinosa@cib.csic.es (M.E.)

**Keywords:** toxin-antitoxin systems, genetic manipulation, gene containment, cell ablation, high expression cell lines, gene therapy, antiviral therapy, anticancer therapy

## Abstract

Toxin-antitoxin (TA) systems are found in nearly all prokaryotic genomes and usually consist of a pair of co-transcribed genes, one of which encodes a stable toxin and the other, its cognate labile antitoxin. Certain environmental and physiological cues trigger the degradation of the antitoxin, causing activation of the toxin, leading either to the death or stasis of the host cell. TA systems have a variety of functions in the bacterial cell, including acting as mediators of programmed cell death, the induction of a dormant state known as persistence and the stable maintenance of plasmids and other mobile genetic elements. Some bacterial TA systems are functional when expressed in eukaryotic cells and this has led to several innovative applications, which are the subject of this review. Here, we look at how bacterial TA systems have been utilized for the genetic manipulation of yeasts and other eukaryotes, for the containment of genetically modified organisms, and for the engineering of high expression eukaryotic cell lines. We also examine how TA systems have been adopted as an important tool in developmental biology research for the ablation of specific cells and the potential for utility of TA systems in antiviral and anticancer gene therapies.

## 1. Introduction: An Overview of Bacterial Toxin-Antitoxin Systems

Toxin-antitoxin (TA) systems are nearly ubiquitous genetic modules in bacterial and archaeal genomes. They generally comprise a pair of genes coding for a stable toxin and its cognate labile antitoxin. Under normal growth conditions, the toxin is prevented from exerting its lethal effect by the antitoxin. However, environmental stresses usually cause a drastic drop in the levels of the unstable antitoxin in the cell due mainly to degradation by endogenous proteases. This leads to activation of the remaining toxin which, in turn, causes either cell death or cell stasis [1,2,3,4,5].

TA systems were initially discovered encoded within bacterial plasmids where they function to mediate plasmid maintenance and stability through the postsegregational killing of any plasmid-free daughter cells that arise within a population. The bacterial hosts became “addicted” to the presence of these TA-encoding plasmids and thus, TA systems were also termed as addiction modules [6,7]. Chromosomal homologues of these plasmid-encoded addiction modules were first reported in *Escherichia coli* and the chromosomal *mazEF* system, comprising the *mazE*-encoded antitoxin and the *mazF*-encoded toxin, was postulated to mediate programmed bacterial cell death under nutrient starvation conditions [8]. This apparent altruistic killing was envisaged to enable part of the bacterial population to survive during adverse conditions, reflecting a multicellular facet of bacteria [8,9,10]. Research into other chromosomal TA systems in *E. coli*, namely the *relBE* system and *chpAIK* (a *mazEF* homologue), appeared to indicate an alternative role. Overexpression of the *relE*- and *chpAK*-encoded toxins in *E. coli* induces a bacteriostatic condition with severe inhibition of translation, but subsequent induction of expression of the respective cognate antitoxins *relB* and *chpAI* fully reverses the toxin-induced stasis [11,12]. *relBE* and other similar TA systems were proposed to function as part of the general stress response of bacteria by regulating the global level of translation and together with the *trans*-translation *ssrA* system, function in the quality control of gene expression [1,13]. However, with increasing numbers of novel TA systems being discovered, their biological functions have expanded, mirroring their genetic diversity. TA systems have been implicated in various other cellular processes such as the formation of persister cells leading to antibiotic tolerance [14,15], as anti-addiction modules [16], in protection against invading bacteriophages [17,18], as stabilization modules for large mobile genetic elements such as superintegrons and genomic islands [19,20], in biofilm formation [21] and in virulence of pathogenic bacteria [22,23,24].

TA systems have so far been broadly classified into five different types, designated types I–V, based on the characteristics of the antitoxin and the mechanisms by which they counteract the toxins [4,5,19]. In type I TA systems, the antitoxin is an antisense RNA that binds to the toxin mRNA, preventing its translation [25]. In type II TA systems, both antitoxin and toxin are proteins and the antitoxin functions by direct binding with the toxin, usually blocking its active site [2]. As for type III TA systems, the antitoxin is an RNA that functions by direct binding, with the toxin protein leading to the formation of a non-lethal protein-RNA complex [26]. In type IV systems, both antitoxins and toxins are proteins but unlike in type II systems, the antitoxins and toxins of type IV systems do not directly interact with each other. Rather, the antitoxin binds to the target of the toxin to prevent the toxin from exerting its lethal effect [27]. Finally, in type V systems, the antitoxin is a protein with ribonuclease activity that cleaves the toxin mRNA and thus prevents the synthesis of the toxin [28]. Nevertheless, a potentially new class of TA system (a possible type VI) was recently discovered in the form of the SocAB system from *Caulobacter crescentus* [29]. Both the SocB toxin and the SocA antitoxin are proteins but in this case, the SocB toxin is the unstable partner due to its susceptibility to the endogenous ClpXP protease. The SocA antitoxin functions as an essential ClpXP adaptor for the SocB toxin, promoting its degradation and thus abolishing its lethality [30]. To date, TA systems belonging to types I and II are the most abundant in prokaryotes with type II TAs being the most well-characterized [5,19].

TA toxins target a wide variety of essential cellular structures and processes such as membrane integrity, cell wall synthesis, DNA replication, ribosome assembly and translation factors, with RNA cleavage being the most prevalent mode of action [3,23]. The near ubiquity of TA systems in prokaryotes and the potential for triggering latent intracellular molecular timebombs, especially in pathogenic bacteria, led to several interesting avenues of research for the use of TA systems as targets for novel antibacterial compounds [5,19,31]. TA systems have also been harnessed as tools in molecular biology, such as for the positive selection of clones containing inserted DNA fragments in cloning vectors. The *ccdB* toxin gene of the *ccdAB* TA system from the *E. coli* F plasmid has been successfully used in a number of cloning vectors where the toxin gene is inactivated upon insertion of foreign DNA, enabling only insert-containing clones to survive and grow [19,32]. With the discovery that some of these bacterial TA systems can be expressed and are functional in eukaryotic cells [33,34,35], several interesting applications have been proposed and developed. In this mini-review, we will look at several strategies used for the heterologous expression of bacterial TA genes in eukaryotic systems and their potential applications in biotechnology and molecular biology.

## 2. Expression of TA Systems in Yeasts: Applications

### 2.1. TA Systems as Tools for Containment in Yeasts

The increasing use of genetically modified organisms (GMOs) in various bioprocesses necessitates appropriate containment strategies to address concerns over the accidental release of these GMOs into the environment, or in cases where the deliberate release of the GMOs into the environment are required for biotechnological applications (such as bioremediation, bioleaching and biopesticides) [36,37]. Two main strategies that have been utilized are passive and active containment systems [36]. In passive containment systems, the GMO is engineered such that it cannot synthesize an essential compound; thus, when these auxotrophic GMOs accidentally escape from the bioreactor or environment where the compound is provided, the organisms will likely die. Recently, “genetically-recoded” bacteria have been engineered whereby the expression of several essential genes is totally reliant on the supply of exogenously supplied synthetic amino acids, resulting in the death of these cells in an environment lacking these synthetic compounds [38,39]. However, such synthetic auxotrophy systems require extensive genome-wide engineering and hence, may not be technologically and economically practical for eukaryotic systems at this point in time. In active containment systems, the GMO is engineered with a controllable lethal function that will not interfere with normal cellular processes until a specific environmental cue is triggered. TA systems have been utilized as the controllable lethal function in several such active containment strategies for bacteria and these have been recently reviewed [36,40].

**Figure 1:**
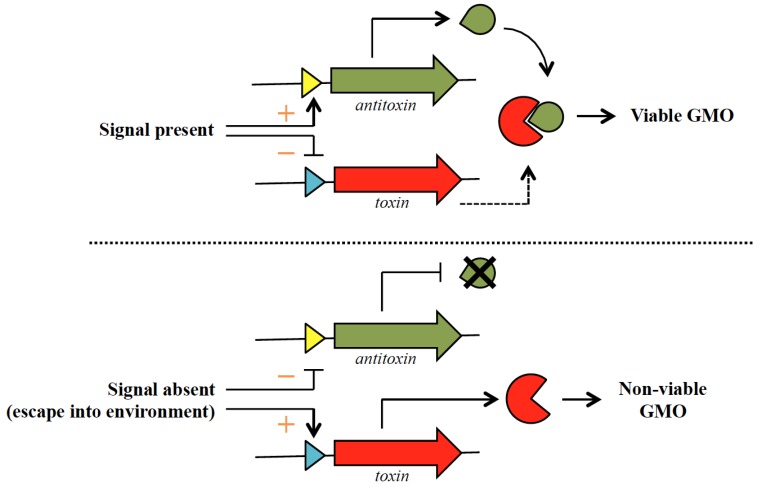
General strategy utilizing a bacterial TA system to engineer a containment system for genetically modified yeasts. The antitoxin-coding gene (depicted by a green arrow) and the toxin-coding gene (red arrow) are cloned under the control of two separate promoters (depicted as yellow and blue triangles) within the same yeast vector. Under controlled conditions (such as in a fermenter), the presence of a specific signal (which can either be a certain media constituent or nutrients such as glucose and specific amino acids), the antitoxin is expressed whereas expression of the toxin gene is repressed (indicated by orange “+” and “−“ signs, respectively). Any leaky expression of the toxin gene (depicted as a dotted arrow) would be countered by the continual expression of the antitoxin. However, in the absence of the specific signal, such as when the genetically modified yeast has escaped into the environment, transcription of the antitoxin gene will be repressed whereas the toxin gene will be actively transcribed, thus killing the escaped organism.

The *E. coli relBE* TA system was demonstrated to be functional in the yeast *Saccharomyces cerevisiae* and was proposed as a containment system for genetically modified yeast [33]. The pYES2 yeast expression vector was used for this purpose with two recombinant plasmids constructed: pKP727 with the *relE* toxin gene under the control of the *GAL1* promoter; and pKP1006 with *relE* controlled by the *GAL1* promoter and the *relB* antitoxin gene under the control of the *MET25* promoter [33]. Expression of *relE* in yeast cells transformed with pKP727 and induced with galactose showed clear inhibitory effects, whereas in yeast cells that were transformed with pKP1006 and induced with galactose in the absence of methionine (for the co-expression of both *relE* and *relB*), higher growth rates were observed, albeit lower than normal [33]. This indicated that the RelE toxin is lethal in *S. cerevisiae* and its toxicity could be somewhat neutralized by co-expression of the RelB antitoxin. However, since that research was published in 2000 [33], there have not been any follow-up reports on the utility of *relBE* or any other TA system for the containment of genetically modified yeasts. A general strategy that could be used to engineer such a TA-based containment system is depicted in Figure 1. Other TA systems have been shown to be functional in *S. cerevisiae* and this includes the *kis-kid* TA system from the *E. coli* plasmid R1 [35] and ε-ζ from plasmid pSM19035 of *Streptococcus pyogenes* [41]. The *kis-kid* genes were expressed in *S. cerevisiae* using a similar system to *relBE* in that the *kis* antitoxin was expressed from a *MET25* promoter (*i.e.*, induced expression in the presence of methionine), whereas the *kid* toxin was under the control of the *CUP1* promoter (induced expression in the presence of Cu^2+^) in an integrative pRS303-derived plasmid recombinant [35]. The ε-ζ genes were, however, expressed by vectors designed for a commercial yeast two-hybrid system (Matchmaker Two-Hybrid System kit from Clontech). Toxicity of the ζ toxin in *S. cerevisiae* was demonstrated, as was its neutralization by its cognate ε antitoxin and interaction between the toxin and antitoxin proteins [41]. The RelE and Kid toxins are endoribonucleases [13,42] whereas the ζ toxin functions by blocking bacterial cell wall synthesis by phosphorylating uridine diphosphate-*N*-acetylglucosamine (UNAG), a key intermediate in peptidoglycan synthesis [43]. In *S. cerevisiae*, the *nucA* gene from the Gram-negative bacteria *Serratia marcescens* has been successfully used to construct a conditional lethal system for containment by placing the *nucA* gene under the control of the glucose-repressed *S. cerevisiae* alcohol dehydrogenase-2 *ADH2* gene promoter [44]. It was proposed that the small size of the *S. marcescens* nuclease (26 kDa) facilitates its entry into the yeast nucleus [44] and it is likely to be the same situation for the smaller ~12 kDa RelE and Kid endoribonuclease toxins. The lethal mechanism for the ζ toxin in yeast is still unknown although it could act to inhibit the synthesis of the yeast cell wall much like in bacteria, as UNAG is a component for the biosynthesis of chitin in yeast [45].

Two new microbial “kill switches” were recently engineered based on the CcdB and MazF TA toxins (along with the *Eco*RI restriction endonuclease) for the containment of genetically modified bacteria. These switches, designated “Deadman” and “Passcode” are modular and flexible (customizable), can be conveniently transferred to other bacterial strains [46] and would therefore be useful in various biotherapeutic and industrial applications. These toxin-based switches should also be functional in yeasts and could likewise be adopted for the containment of genetically modified yeasts.

### 2.2. TA Systems as Tools for the Genetic Manipulation of Yeasts

Expression of another endoribonuclease toxin, MazF, from the tightly controlled, methanol-inducible *AOX1* promoter in the methylotrophic yeast *Pichia pastoris*, was found to be lethal. This was used as the basis of a novel method for unmarked genetic modification of *P. pastoris* using *mazF* as a counter-selectable marker [47]. A modular plasmid, pKSCTMF, was constructed consisting of an *AOX1* promoter-*mazF* expression cassette and a Zeocin-resistance gene as a dominant selection marker flanked by direct repeats from segments of the *CYC1* transcriptional terminator (TT), as recombination sites, along with multiple restriction sites to permit subcloning of fragments homologous to the *P. pastoris* genome for disruption [47]. *P. pastoris* was first transformed with a linearized recombinant pKSCTMF construct and selected on medium supplemented with Zeocin. Transformants with the correct disruption were determined by phenotype and/or PCR analysis. Subsequently, selection for recombination of the two *CYC1* TT repeats was performed by growing the transformants on methanol medium plates that would enable only transformants with a single *CYC1* TT segment inserted into the disrupted site to survive [47], *i.e.*, effectively disrupting the gene of interest and subsequently evicting the selectable markers. This would enable the disrupted strains to be amenable for a second or multiple genetic modifications using the same MazF-ZeoR cassette (marker recycling). This method also enabled knock-in of a gene of interest as well as site-directed mutagenesis at the native locus [47].

A variant of this method was utilized to enable disruption of certain genes that were less amenable to gene replacement in *P. pastoris* such as *OCH1* that encodes α-1,6-mannosyltransferase and for which gene replacement occurs at frequencies of <1% even with flanking arms that are longer than 1 kb [48]. Here, the *AOX1* promoter-*mazF* cassette was cloned into an episomal “helper” plasmid that also contained a full copy of the *OCH1* gene, providing a functional backup of the *OCH1* gene for *P. pastoris* before deletion to avoid compromising the fitness of the yeast cells due to possible loss of function. *P. pastoris* carrying this helper plasmid was then used as the host for conventional *OCH1* gene disruption using a Zeocin-resistance gene [48]. Once replacement of the chromosomal *OCH1* gene with the Zeocin-disrupted copy had been validated, these strains were grown in medium containing methanol to induce expression of *mazF*. This exerts a strong selection pressure for the strains to lose the episomal “helper” plasmid. The same strategy was used to successfully delete the *KU70* and *SGS1* genes in *P. pastoris*, and increasing their targeting efficiencies [48].

## 3. TA Systems as Cell Ablation Tools

### 3.1. Cell Ablation for the Containment of Genetically Modified Plants

Transgenic crops have become integrated into modern agriculture and are increasingly adopted worldwide. Besides having useful traits such as herbicide resistance and pest tolerance, transgenic plants have also served as a platform for the large-scale production of recombinant pharmaceutical proteins (also known as biopharming) and industrial enzymes [49]. One of the major concerns of the wide-scale adoption of transgenic crops is their accidental spread into the environment leading to contamination of the human food chain. This concern is not without precedent as several episodes of accidental release or contamination have occurred, leading to a negative perception of GM crops in the eyes of the public [49,50]. In addition to physical containment methods, biological containment strategies such as plastid transformation, male sterility and genetic use restriction technologies (GURTs) have been proposed or developed to confine the potential spread of transgenic crops [51,52,53]. Engineered sterility of transgenic plants is not only helpful in preventing their pollen spread into natural ecosystems, it is also useful for the development of male sterile parents for hybrid seed production [54] in self-pollinated crops, for the removal of allergenic pollen and for the production of seedless varieties of fruit and vegetable crops [55]. To achieve sterility, a toxin gene is placed under the control of a tissue-specific promoter that enables the toxin to be expressed in certain parts of the plant anther and/or pistil.

One of the earliest and successfully applied bacterial toxin genes that has been used for the specific ablation of the plant’s reproductive organs leading to sexual sterility is Barnase, a small 12.4-kDa RNase of 110 amino acid residues produced by *Bacillus amyloliquefaciens* that also synthesizes its antidote, a small protein called Barstar (89 amino acid residues) that binds to Barnase and sterically blocks the Barnase active site in a one-to-one noncovalent interaction [56]. Barnase has been used since the 1990’s to engineer male, female or bisexual sterility in various transgenic plants by placing the *barnase* gene under the control of a tissue-specific promoter, which enables it to be expressed in certain parts of the plant anther and/or pistil [56,57,58,59,60,61]. Expression of *barnase* leads to the destruction of the reproductive organs, thereby conferring sterility to the transgenic plants. Although not a TA system, Barnase-Barstar is illustrative of the utility of a bacterial regulatory module where the toxic effect can be rescued by the specific counterpart; in the case of sexually sterile (*barnase* expressing) plants, this has been applied by the expression of *barstar* in “fertility restorer” plant lines, when viable pollen or seed is required for hybrid seed production [62]. Unlike in TA systems, the genes for Barnase and Barstar are not in an operon and are located in different loci in the *B. amyloliquefaciens* genome, suggestive of distinct regulation of their expression [56]. However, this is not a barrier to similar application of TA systems, since the toxin and antitoxin can be expressed as independent units in a transgenic context and in fact this permits differing expression levels, for example, stronger expression of the “restorer” molecule when required [62]. Other bacterial or bacteriophage toxins or enzymes that have been applied for negative selection in plants include the expression of the restriction endonuclease *Eco*RI in tobacco pollen [63] and cytosine deaminase from *E. coli* and the dipththeria A-chain toxin (*DT-A*) subunit from corynephages of *Corynebacterium diptheriae* for gene targeting in rice [64].

**Figure 2:**
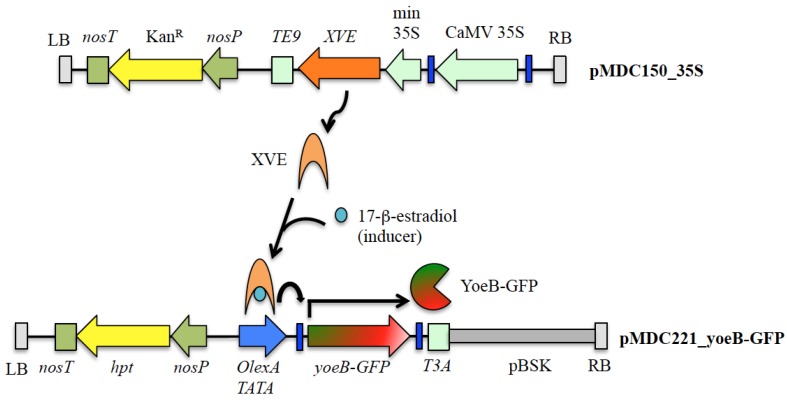
Schematic diagram illustrating the Gateway-compatible two-component inducible expression system used by Abu Bakar *et al.* [65] for the expression of the YoeB_Spn_ toxin from *Streptococcus pneumoniae* in the model plant *Arabidopsis thaliana*. The genetic organization of the T-DNA regions flanked by the left border (LB) and right border (RB) of the recombinant activator vector, pMDC150_35S and the recombinant responder vector, pMDC221_yoeB-GFP, are shown. The cauliflower mosaic virus (CaMV) 35S promoter was cloned in between the Gateway *attR* recombination sites (indicated in dark blue boxes) of the pMDC150 activator vector enabling the constitutive expression of the XVE chimeric transcriptional activator in transgenic plants. The *yoeB_Spn_* toxin gene was cloned as a translational fusion with the green fluorescent protein gene, *GFP* in between the *attR* recombination sites of the responder vector pMDC221, thus placing the *yoeB-GFP* fusion gene under the control of the XVE-responsive promoter (OlexA-TATA). Both pMDC150_35S and pMDC221_yoeB-GFP were co-transformed into *A. thaliana* via *Agrobacterium tumefaciens*-mediated transformation [65]. Transcription of *yoeB-GFP* in transgenic *A. thaliana* only occurs in the presence of the inducer 17-β-estradiol that activates the XVE activator. The vectors also contain the *nos* promoter (*nosP*) to drive the expression of the kanamycin resistance gene (Kan^R^) in pMDC150 and the hygromycin resistance gene (*hpt*) in pMDC221 for plant selection. The pMDC221 T-DNA also contains the pBluescript vector sequence (pBSK; grey rectangle), which can be used for plasmid rescue procedures due to the presence of the ampicillin resistance gene and the ColE1 origin of replication, which enables replication in *E. coli* [66]. *TE9*, *TE9* terminator; *T3A*, terminator; *nosT*, *nos* terminator.

The variety of toxin targets for bacterial TA toxins gives researchers a choice of toxins to use for specific cell ablation. Like Barnase-Barstar, each of these TA toxins are available with their corresponding antitoxins, which can be used to modulate the expression of the toxin to avoid excessive levels of the toxin, which may spread to other tissues causing undesirable effects. Transgenic *Brassica napus* plants with *barnase* being ectopically expressed from a seed myrosin cell-specific *Myr1.Bn1* promoter were found to be embryo lethal. Co-expression of *barstar* from a constitutive cauliflower mosaic virus (CaMV) 35S promoter enabled selective and controlled death of myrosin cells without affecting plant viability [67]. Until now, only the YoeB_Spn_ endoribonuclease toxin from the YefM-YoeB_Spn_ TA system of the Gram-positive human pathogen *Streptococcus pneumoniae* [68] has been demonstrated to be functionally lethal in the model plant *Arabidopsis thaliana* [65] and our unpublished results indicated that co-expression of the cognate YefM_Spn_ antitoxin was able to abrogate the lethality of YoeB_Spn_ in *A. thaliana*. A two-component XVE-based inducible expression system was used to assess the functionality of the YoeB_Spn_ toxin in *A. thaliana* (Figure 2) [65]. XVE is a chimeric transcription activator comprising of the DNA-binding domain of the LexA bacterial repressor, the transactivating domain of VP16 and the *C*-terminal region of the human estrogen receptor ER and is strictly activated by estradiol in transgenic plants [69]. The *yoeB*_Spn_ transgene was cloned as a translational fusion with the green fluorescent protein (GFP) gene under the control of the XVE-responsive promoter (consisting of the LexA operator sequence fused to a 35S minimal promoter) [65,66]. Activation occurs when the inducer 17-β-estradiol binds to the XVE activator, enabling it to bind to the LexA operator sequences and thereby activating transcription of the cloned transgene. Plant defects and tissue necrosis were observed in 17-β-estradiol-induced transgenic *A. thaliana* expressing the YoeB_Spn_-GFP fusion 3 days after induction followed by plant death over a period of 9 days [65]. The system is tightly regulated with no detectable transactivating activity in the absence of an inducer [66,69]. Hence, despite containing a cytotoxic gene such as the dipththeria A-chain toxin (*DT-A*) [66] and YoeB_Spn_ [65], transgenic plants developed normally in the absence of the inducer. At this juncture, the use of YoeB_Spn_ or any other bacterial TA toxins for the ablation of specific plant cells has yet to be carried out. Nevertheless, the potential is there and we are currently exploring the possibility of developing male sterile plants by using tissue-specific promoters to express the *yoeB*_Spn_ toxin gene.

### 3.2. Cell Ablation in Developmental Biology Research of Higher Eukaryotes

The *E. coli* plasmid R1-encoded *kis-kid* TA system was shown to be functional in yeast [35]. To investigate its functionality in higher eukaryotes, purified TA proteins were microinjected into embryos of the frog *Xenopus laevis* as well as the human cell lines HeLa and SW480 [35]. Injection of the Kid toxin into two-cell embryos of *X. laevis* led to failure of the Kid-injected blastomere to develop normally, unlike blastomeres that were injected with a combination of Kid and its antitoxin Kis. The Kid-injected half embryo showed very few cells, most of which were anucleated [35]. The effect was equally lethal in human cell lines, as microinjection of Kid into HeLa and SW480 cells drastically decreased their survival and eventually led to their death. The lethality of Kid was completely abrogated when Kis was preincubated with Kid prior to microinjection [35]. Ablation of a specific cell type in a developing organism using the Kis-Kid TA system was subsequently successfully demonstrated for zebrafish (*Danio rerio*) in targeting primordial germ cells [70]. One-cell-stage embryos were injected with mRNA encoding for *kid* fused to the 3′-UTR of the zebrafish *nos1* gene, which directs expression of the Kid toxin preferentially to the primordial germ cells. The treatment effectively eliminated the primordial germ cells and resulted in somatic defects and embryonic death due likely to leaky expression of *kid* in other cells [70]. To protect the somatic cells from the lethal effects of *kid* expression, the mRNA of the *kis* antitoxin gene fused to the *globin* 3′-UTR was co-injected along with the *kid*-*nos1*-3′-UTR mRNA. These embryos showed primordial germ cell loss but appeared morphologically normal and could be raised to adulthood without any somatic defects. Interestingly, all the germ cell-ablated embryos developed as sterile male adult fish that were capable of inducing females to lay eggs but not in fertilizing the eggs due to undeveloped gonads. It was thus concluded that in zebrafish, the germ line is essential for the development of females but is dispensable for the development of male somatic tissues with the exception of the gonad [70]. The findings also demonstrated that TA proteins could be applied for highly specific ablation of targeted eukaryotic cells, hence a potentially important tool in developmental studies.

The *kis-kid* genes were also cloned into expression vectors and transfected into human cell lines to investigate whether independent transcriptional control of the TA genes would enable regulated cell killing or survival in human cells [35]. In one of the recombinant constructs, the *kid* toxin gene was placed under the control of the constitutive cytomegalovirus (CMV) early promoter while transcription of the *kis* antitoxin gene was controlled from a tetracycline-repressible promoter in which the presence of the tetracycline analogue, doxycycline, would lead to transcriptional repression in HeLa TetOff cells [35]. The *kis* and *kid* genes were cloned in a tail-to-tail orientation. When Kid was expressed without Kis in the transfected HeLa TetOff cell lines (*i.e.*, in the presence of doxycycline), cell death was widespread beyond three days, with total cell death after 15 days. Such a lethal effect was not observed in the absence of doxycycline (*i.e.*, co-expression of Kid and Kis), indicating that inhibition of cell proliferation can be modulated in human cells through independent transcriptional control of *kis* and *kid* [35]. Similar observations were reported earlier for experiments conducted with the *E. coli*-encoded RelE toxin in a human osteosarcoma cell line, TREx-U2OS [34]. In this case, the *relE* gene was cloned under the control of a Tet promoter-operator and in the TREx-U2OS cells, the Tet repressor is constitutively expressed. Hence, in the transfected TREx-U2OS cells, expression of *relE* is induced by addition of tetracycline. Expression of RelE was indeed detrimental to cell growth with estimation of less than 1 in 10^8^ cells surviving the expression of the toxin and metabolic activity as measured using the MTT assay, showing an obvious decline 12 h after tetracycline induction [34]. However, Yamamoto *et al.* [34] did not show if co-expression of the cognate RelB antitoxin was able to counteract the toxic effects of RelE expression. The authors did indicate that cells that were induced for RelE expression showed morphological changes that are characteristic of apoptosis such as membrane budding, reduction in cell volume, chromatin condensation and fragmentation. DNA laddering, typical of caspase-activated DNase was also shown [34]. HeLa cells expressing the Kid toxin were also demonstrated to undergo apoptosis through propidium iodide and Annexin-V staining, which are early markers of apoptosis, along with the characteristic morphological changes [35]. A more extensive study was carried out by Shimazu *et al.* [71], who showed that expression of the *E. coli* MazF toxin in human T-Rex-293 cells resulted in cellular mRNA degradation, inhibition of protein synthesis and induction of apoptosis with activation of the caspase-3 executioner caspase. The pathway that was utilized to activate apoptosis triggered by MazF-induced mRNA cleavage, was also elucidated resulting in the identification of the BH3-only proapoptotic protein NBK/BIK as a mediator of apoptosis induced by adenovirus infection [71].

## 4. TA Systems as Tools for Overproduction of Heterologous Proteins in Eukaryotic Cells

The strength and stability of transgene expression in transfected mammalian cell lines depends mainly on the chromosomal integration site, which occurs mostly at random. A lot of time and effort is required to screen and identify stable transfected clones that highly overexpress the gene of interest [72]. Nehlsen *et al.* [73] developed a method that utilized the Kis-Kid TA system to enable a more efficient selection and enrichment of mammalian cells that highly express recombinant genes. The initial step in this strategy is to stably establish in transfected CHO-K1 cells the ability for controlled expression of the *kid* toxin gene, which was placed under Tet-dependent transactivation control. Cells were cultivated in the presence of doxycycline (Dox) to protect the cells from the lethality of Kid. Cell clones were evaluated for viability of growth in the presence and absence of Dox with clones selected that showed normal growth in the presence of Dox and at least 80% cell death when grown without Dox for 10 days [73]. These cells were then transfected with a plasmid from which the gene of interest and the *kis* antitoxin gene were transcriptionally coupled using an internal ribosome entry site (IRES) that enabled a bicistronic arrangement in eukaryotic cells. Expression data from three different transgenes (luciferase, eGFP and an IgG antibody, the genes of which were driven by an SV40 promoter), showed that transfectants that expressed the Kid toxin steadily increased their transgene expression over several weeks (up to 100-fold increase for the IgG antibody), whereas in the absence of toxin expression, transgene expression in the transfectants dropped over the same period of time [73]. The increased expression within the pools of *kid*-transfected cells was possibly due to a selection process for highly expressing clones created by random integration of the transgene-*kis* cassette. Cells with reduced expression levels are likely eliminated or are over-grown. Thus, to apply this system in other cells, the lethal effect of *kid* or any other TA toxin expression needs to be validated in the cell line of interest. Furthermore, coupled expression of the gene of interest with the *kis* or other antitoxin genes must be achieved [73].

## 5. TA Systems in Gene Therapy

### 5.1. Antiviral Gene Therapy

The development of TA systems for application in antiviral gene therapy is seemingly feasible, as has been demonstrated in several elegant studies, and strategies to employ them as possible drugs were thoroughly described previously [31]. As most of the toxins of TA systems are ribonucleases, this would have potential in particular for the control of RNA viruses. To exploit TA systems as antivirals, specificity has become a major concern, as while bacterial endoribonuclease toxins usually cleave at specific RNA sequences or sites, they are not cell-specific. Activation of toxin effect through a viral promoter in response to its specific protein, and activation through cleavage by a specific viral protease, are two rather clever approaches to explicitly tackle the viral-infected cells. The first approach is exemplified by the Tat (transactivator of transcription) protein, which is a viral regulator protein produced during the early stage of HIV-1 infection by HIV-1 viruses. The Tat protein is essential in HIV infection as HIV-1 mutants lacking a functional *tat* gene are not able to proliferate. The Tat regulator protein will bind to the specific long terminal repeat (LTR), termed the transactivation response (TAR) sequence, that consequently induces the production of various HIV-1 proteins for infection purposes [74]. Thus, therapy using a construct with a toxin gene placed downstream of a TAR sequence in a retroviral vector, during the early stage of HIV-1 infection, should result in activation of expression of the toxin gene upon the binding of the Tat regulator protein to the TAR promoter sequence. MazF is one of the most well-studied TA system toxins, and the *E. coli*-encoded MazF functions as an endoribonuclease that cleaves mRNA specifically at ACA codons [75]. The *E. coli mazF* gene itself harbors nine ACA codons, and can be engineered to be void of ACA sequences without altering the amino acid sequences, to prevent self-cleavage and yet maintain the toxic effect. As the HIV RNA contains over 240 ACA sequences, it is thus a very good target for MazF. It was shown that when a human T lymphoid line CEM-SS, that is highly prone to HIV infection, was transduced with an HIV-1-LTR-regulated MazF recombinant plasmid and then infected with HIV-1 IIIB, the replication of the virus was thwarted, as HIV-1 IIIB p24 could not be detected in the culture medium [74]. In addition, the CD4 level was also not affected. Fortunately, although MazF was also able to cleave cellular mRNA, the levels of induced MazF did not seem to cause serious cell damage and thus normal cellular growth was maintained. Similar results were also observed in an experiment whereby the Tat-dependent MazF expression system was performed in rhesus macaque primary CD4+ T cells from monkeys that were infected with the suppressed chimerical virus simian/human immunodeficiency virus SHIV 89.6P [74].

In general, the shift of a patient from chronic phase to AIDS phase is caused by the continuous growth of the HIV virus and the body’s suppressed immune system that fails to protect the infected cells, which subsequently lead to decreases in CD4+ T cells. Therefore, the inhibition of viral growth by the MazF-based therapy to control and protect the cells from HIV viruses and to maintain the immune system is somewhat important. To examine the persistence and the *in vivo* safety of the MazF-transduced autologous CD4+ T cells (herein called MazF-Tmac cells), cynomolgus macaque primary CD4+ T cells were first transduced with the HIV-1-LTR-regulated MazF recombinant plasmid, then infused into the autologous monkeys, and several parameters were monitored for more than half a year [76]. As a result, even though the levels of MazF-Tmac cells in the peripheral blood gradually decreased, their level was still significantly detected throughout the entire experimental period and MazF-Tmac cells were also detected in the lymphoid tissues and the spleen. Strikingly, no lesions were observed and antibodies against MazF were also not detected. Moreover, the gene-modified cells harvested from the monkeys more than half a year post-infusion were still able to inhibit the replication of SHIV 89.6P [76]. These combined results reflect the persistency and safety of this promising MazF-based anti-HIV virus approach.

Besides Tat-dependent activation of the MazF toxin, another interesting approach made use of viral proteases, which cleave at specific cleavage sites, to activate the toxin to explicitly target the infected cells. A non-structural serine protease (NS3)-activated MazF system was constructed, in which NS3-4A is a hepatitis C virus (HCV) protein that is important in the replication of HCV. The NS3-activated MazF construct was designed by fusing the NS3 protease cleavage site in between MazF and the truncated *C*-terminal of MazE, which is the cognate antitoxin of MazF, and thus the resulting products neutralize the toxic effect of MazF. MazF can be activated by incubating the inert proteins with NS3 protease that cleaves the linker in between the MazE and MazF proteins, thereby releasing the MazF toxin [77]. The same principle applies to other viral proteases such as HIV-1 protease and factor Xa [77]. When a similar construct of the NS3-activated MazF system, termed “zymoxin”, was placed in HEK293 T-REx cells that harbored the tetracycline-inducible NS3–4A constructs, NS3-mediated activation of MazF that inhibited cellular protein synthesis was observed. However, unlike the MazF-Tmac cells, cytotoxic effects were observed although this was with low levels of NS3 [78]. Thus, the dosage of MazF needs be fine-tuned to eliminate its harmfulness to human cells to make this antiviral approach more feasible.

### 5.2. Anticancer Gene Therapy

Although TAs are absent in eukaryotic cells, TA toxins have been found to inhibit growth in *Saccharomyces cerevisiae* and *Arabidopsis thaliana* (see above). More interestingly, these toxins were able to trigger apoptosis in human cells [34,35]. This finding has opened new avenues to explore the feasibility of using TAs as tools to develop anti-tumor drugs [79,80]. The hypothesis was based on the finding that the toxins Kid from plasmid R1 [81], and MazF from the chromosome of *E. coli* (reviewed by [82]) were able to induce apoptosis in eukaryotic cells. It was found that induction of apoptosis by MazF toxin in human cells was dependent of the BAK pro-apoptotic protein and its upstream regulator NBK/BIK; cells defective in BAK, however, did not exhibit apoptosis but MazF was still able to cause total inhibition of protein synthesis [71]. Even though not many studies on the activity of prokaryotic toxins on oncogenic cells have been reported, it has been recently shown that toxins VapC 22 (from the chromosome of *Mycobacterium tuberculosis* H37Rv), and PasB (from plasmid pTF-FC2 from *Thiobacillus ferroxidans*) exhibit pro-apoptotic activity in diverse human cancer cell lines [83]. However, most of these studies have been conducted using cell-cultures (*i.e.*, under *in vitro* conditions) transfected with plasmids harboring genes encoding the bacterial toxin [84]. Then, there are a number of remaining questions to be tackled, such as how to target cancer cells with TAs while avoiding potential harmfulness to the normal cells? Are there activator-regulatory proteins present in oncogenic cells that could be used to mimic the antiviral therapy mentioned above? And last, but not least, what ways can be envisaged to deliver the desired anti-cancer toxin into tumor cells and not into healthy cells?

**Figure 3:**
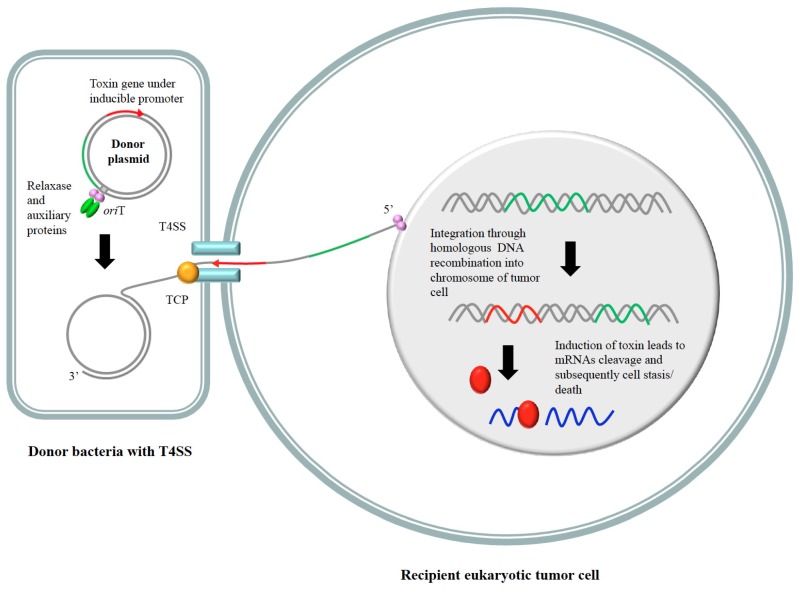
Possible way to deliver an RNase toxin into the chromosome of a tumor cell. The plasmid-encoded relaxase (depicted in pink) harbors a toxin gene (denoted as a red arrow), and a DNA stretch homologous to the chromosome of the recipient cell (shown as green lines). The relaxase recognizes and initiates transfer from the donor (bacterial) cell by means of the protein-DNA complex formed by the relaxase, auxiliary proteins (green), and *oriT* (shadowed). This complex is pumped into the recipient tumor cell by the coupling protein (CP, shown in orange) and the T4SS export system (in blue). The relaxase-DNA enters into the nucleus and integrates its cargo transgene into the chromosome of the recipient cell. Induction of the toxin leads to RNA cleavage and tumor cell stasis.

Stable expression of a foreign gene integrated within the chromosome of a mammalian cell would depend greatly on a number of factors, but especially on the region where integration occurred, on the surrounding DNA context (adjacent genes, DNA structure), and on the promoter used to express the transgene. In many instances, integration occurs randomly if no targeted site has been chosen. This makes it very tedious and difficult to detect the cells that have integrated the transgene. Consequently, a targeted integration site would be generally favored. An excellent and imaginative approach to tackle the above questions and to achieve stable expression of heterologous genes in eukaryotic cells is proposed using the *Agrobacterium tumefaciens* Ti plasmid machinery, or any other T4 secretion system (T4SS) protein complex, to specifically integrate the desired genetic information within any eukaryotic chromosome [85]. T4SS is used by bacteria to translocate and transport DNA-protein complexes from a donor to a recipient cell (Figure 3). This strategy has been used to develop gene cassettes that make use of the site-specific recombinase and integrase ability of some bacterial plasmid-encoded relaxases (proteins devoted to conjugative transfer between bacteria or between bacteria and eukaryotes). In some cases, it has been shown that the target sequence for the relaxase to perform a strand transfer reaction could be as short as 17 nucleotides, and furthermore, the relaxase can integrate the transferred DNA into the nucleus of eukaryotic cells if it finds sequences homologous to its target in the recipient cells [86]. We speculate that the relaxase activity could be also used to efficiently integrate the desired genes into eukaryotic chromosomes, provided some stretches of homologous DNA are cloned within the incoming plasmid DNA [86,87]. Employment of these cassettes is predicted to function in an efficient manner for gene therapy with specific targeting into the recipient chromosome, rather than random integration [88]. If the incoming plasmid harbors a toxin-encoding gene and some specific tumor-related DNA region, we could envisage that, at least as a preliminary experimental approach, integration of the toxin gene into the desired chromosome region of tumor cells would be feasible. If, in addition, the toxin gene is cloned under the control of any oncogenic-specific promoter, or any inducible promoter that can be used in eukaryotic cells, then we could also consider that some targeted-vectors could be used to employ toxins as specific anti-cancer tools.

## 6. Conclusions

Our knowledge of bacterial TA systems has improved tremendously over the past two decades since their initial discovery in bacterial plasmids and then, bacterial chromosomes, where they were postulated to mediate programmed bacterial cell death [8]. These small genetic modules are now known to be almost ubiquitous in bacterial and archeael genomes and to play essential roles in diverse cellular processes [2,3,89]. As we have shown in the above review, the finding that TA systems are functional in eukaryotic cells has opened the door to various innovative biotechnological and biomedical applications. With the inevitable growth in our fundamental knowledge of TA systems and with more novel TA systems being discovered, further refinements to existing applications will be seen and even more interesting and novel applications will be presented. TA systems have indeed came a long way since the time when they were viewed as small, curious genetic entities that helped to maintain the stability of bacterial plasmids to their present position as one of the important tools in the toolbox used in the current on-going biotechnology revolution.

## Abbreviations

The following abbreviations are used in this manuscript:

CMV: cytomegalovirus
GMO: genetically-modified organism
HCV: hepatitis C virus
HIV: human immunodeficiency virus
IRES: internal ribosome entry site
TA: toxin-antitoxin
T4SS: type IV secretion system
UNAG: uridine diphosphate-*N*-acetylglucosamine
